# Cell Compartment is a Predictor of Protein Rate of Evolution, but not in the Manner Expected: Evidence Against the Extended Complexity Hypothesis

**DOI:** 10.1093/gbe/evaf126

**Published:** 2025-06-21

**Authors:** Juan Rivas-Santisteban, Pablo Yubero, Laurence D Hurst

**Affiliations:** Systems Biology Department, Centro Nacional de Biotecnología (CNB-CSIC), Madrid, Spain; Milner Centre for Evolution, Department of Life Sciences, University of Bath, Bath, UK; Systems Biology Department, Centro Nacional de Biotecnología (CNB-CSIC), Madrid, Spain; Milner Centre for Evolution, Department of Life Sciences, University of Bath, Bath, UK

**Keywords:** protein evolution, subcellular location, population genetics, extended complexity hypothesis, comparative genomics

## Abstract

What accounts for the variation between proteins in their rate of evolution per synonymous substitution (i.e. d*N*/d*S*, alias *ω*)? Previous analyses suggested that cell location is predictive, with intracellular proteins evolving slower than membrane proteins, a result considered supportive of the extended complexity hypothesis. However, as they occur in 3D space, cytoplasmic proteins are expected to be more abundant. As the level of gene expression is the strongest predictor of *ω*, and many predictors of protein rate variation are explained by covariance with it, here we ask whether the cell compartment effect is explained by covariates. We employ two single-celled species for which there exist exceptional data, the bacterium (*Escherichia coli*) and the eukaryote (*Saccharomyces cerevisiae*). For both, we establish informative species trios to determine branch-specific *ω* values. In both species, in the absence of covariate control, cytoplasmic proteins evolve relatively slowly, while membrane proteins evolve fast, as originally claimed. After controlling for protein abundance, however, membrane proteins have the lowest rates, this inversion being resilient to multiple alternative abundance control methods. The effect size of the cell compartment as a predictor is of a comparable magnitude to the essentiality effect and remains when allowing for essentiality. We conclude that the effects of the cell compartments are real, but their direction is dependent on the presence or absence of abundance control. These results question any model, such as the extended complexity hypothesis, that claims support from a slower evolution of cytoplasmic proteins and underscore the importance of covariate control.

## Graphical Abstract

**Graphical Abstract:**
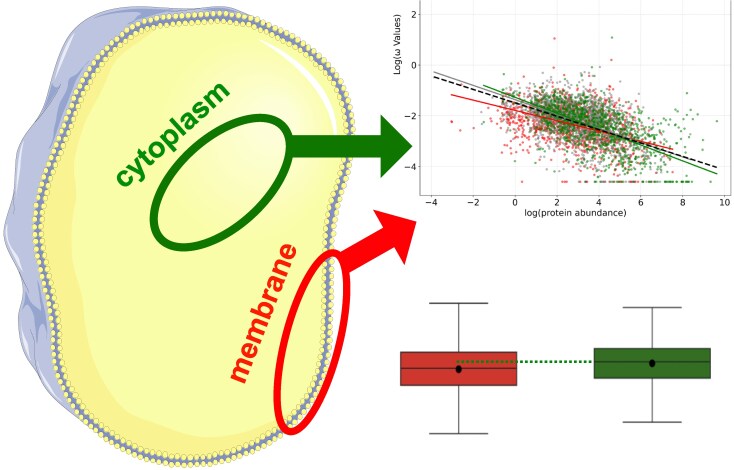

Significance There are a few synthetic frameworks that try to explain why proteins vary in their rate of evolution. The extended complexity hypothesis postulates that proteins functioning within more complex cell systems will evolve slower and claims for support the observation that proteins that function at the periphery of cells evolve relatively fast. However, we find that, controlling for protein abundance, the reverse is true. These results question any model that claims support from a slower evolution of cytoplasmic proteins and underscore the importance of covariate control.

## Introduction

Proteins vary considerably in their rate of protein evolution per synonymous site substitution, known variously as d*N*/d*S*, $K_{a}/K_{s}$, or omega (*ω*) (Pál et al. 2006). Why this is, is however less clear and has been an ongoing concern within molecular evolution circles (Kimura 1987; Kreitman 1996; Pál et al. 2006; Akashi et al. 2012; Zhang and Yang 2015). Obvious structural constraints, such as the density of functional motifs, explains a good proportion of the between-protein variance (Wolf et al. 2008). Many other factors, such as degree to which they function as hubs (Fraser et al. 2002, 2003; Jordan et al. 2003; Batada et al. 2006; Biswas et al. 2018), the knockout fitness (Hurst and Smith 1999; Yang et al. 2003), singleton/duplicate status (Lynch and Conery 2000; Van de Peer et al. 2001; Magadum et al. 2013; Pegueroles et al. 2013; Woods et al. 2013; O’Toole et al. 2018), pleiotropy (Zeng and Gu 2010), protein conformational diversity (Echave et al. 2016), or gene compaction/size of exons (Liao et al. 2006; Shin and Choi 2015) have been considered as correlates. However, few strong and robust correlates have been observed. In mammals, constraints on exon ends to enable efficiency splicing result in exon size being one such strong predictor (Parmley et al. 2007). Nevertheless, a universal strong predictor is the degree to which the protein is expressed (Duret and Mouchiroud 2000; Pál et al. 2001; Subramanian and Kumar 2004). Indeed, many correlates disappear, or are substantially diminished in importance, when controlling for expression level (Bloom and Adami 2003, 2004; Pál et al. 2003; Rocha and Danchin 2004; Batada et al. 2006, 2007).

While there are then many potential predictors, there are fewer attempts to provide a broad synthetic view of protein evolution. One attempt focuses on problems resulting from processing errors (mistranslation, misfolding, and misinteraction), this being proposed to explain why highly expressed proteins evolve slowly (Drummond and Wilke 2008; Zhang and Yang 2015). However, the extent to which misfolding explains protein rates of evolution is now contested (Plata and Vitkup 2018; Usmanova et al. 2021), and simpler models relating higher dose to higher dose change sensitivity suggest that evoking error-prone folding may be unnecessary (Cherry 2010; Gout et al. 2010). An alternative synthetic view is proposed by the extended complexity hypothesis (Aris-Brosou 2005), hereafter ECH. The original complexity hypothesis (Jain et al. 1999) addressed the problem of why some genes are more likely to be horizontally transferred than others, arguing that those more interconnected, more embedded in complex systems, are harder to successfully establish in a new genome. Interconnectedness can here be defined in multiple dimensions: protein interactions, complexity of networks within which protein activity is embedded, etc. The ECH applies similar logic to explain why some proteins are slower evolving than others (Aris-Brosou 2005). The broad prediction is that highly interconnected proteins should be more constrained. Extending the observation that extracellular proteins evolve unusually fast (Winter et al. 2004; Julenius and Pedersen 2006; Dean et al. 2008; Nogueira et al. 2012), the ECH takes as support a trend for faster evolution of proteins working at the cell periphery (membrane proteins, extracellular, etc., Aris-Brosou 2005) (see also Julenius and Pedersen 2006; Sojo et al. 2016). The presumption is that the complex systems are more physically central in a cell (transcription, translation, replication, etc.), while membrane proteins, for example, tend to function more as islands in the sea of membrane.

A priori, however, we might expect faster evolution of proteins on membranes if only as a covariate to expression level. Membranes can be regarded as two-dimensional (2D) structures and so the abundance of membrane proteins may be expected to be lower than that of, for example, cytosolic proteins (Engelman 2005) as these operate in 3D space. In numerous other instances claims that certain variables explained protein rates of evolution turned out to reflect little more than covariance with expression level (Bloom and Adami 2003, 2004; Pál et al. 2003; Rocha and Danchin 2004; Batada et al. 2006, 2007), including the number of protein–protein interactions (Bloom and Adami 2003, 2004), a result previously considered supportive of the ECH (Aris-Brosou 2005). From an evolutionary point of view, then, the more interesting question would be whether any differences in rates of evolution between cytoplasmic and membrane proteins are explained as a consequence of protein abundance. For the general case expounded by the ECH, this was not considered. In the case of extracellular versus intracellular proteins, there is ambiguity as to whether this is explained by covariates such as expression level and essentiality (Julenius and Pedersen 2006; Liao et al. 2010).

Here, then, we return to these issues employing presently available exceptional resources for two single-celled species, *Escherichia coli* and *Saccharomyces cerevisiae*, both of which are expected to have efficient selection as they have historically large effective population sizes. Covariate analyses of expression level commonly rely on proxies to protein abundance, notably RNA transcript level, which is known to sometimes be a poor proxy (Maier et al. 2009). Not only can we define cell location for proteins in *E. coli* and *S. cerevisiae* but, in addition, both are now very well represented in PaxDB (Wang et al. 2015), the inventory of protein abundance measures. The integrated datasets cover 91% and 96% of all protein-coding genes in *E. coli* and *S. cerevisiae*, respectively. We can thus also expand the taxonomic coverage of prior analyses that have to date considered eukaryotes alone. We thus ask in *E. coli* and *S. cerevisiae* (a) whether the protein rate of evolution is heterogeneous between cell compartment classifications, (b) whether protein abundance is heterogeneous between cell compartment classifications, and (c) whether any variation in protein abundance explains the between-compartment variation in rates of protein evolution. In addition, we ask whether essentiality/dispensability might explain any effects, as suggested for extracellular proteins (Julenius and Pedersen 2006; Liao et al. 2010).

We find that there is heterogeneity between cell compartments in uncontrolled rates of protein evolution with cytoplasmic proteins evolving slowly, as previously claimed (Aris-Brosou 2005; Sojo et al. 2016). However, the direction of the effects reverses on control for protein abundance. Membrane proteins thus have either relatively slow or fast evolution dependent on whether we control for protein abundance or not. The cell compartment explains approximately as much variation in rates of evolution as does dispensability and remains a predictor after control for dispensability. This renders it a modest, but probably real, predictor. These results argue against the ECH and provide a unique (to the best of our knowledge) instance where the controlled and uncontrolled results provide inverted results, rather than just a diminution of effect size, rendering the abundance control all the more important.

## Results

### In *E. coli* and *S. cerevisiae* Cytoplasmic Proteins are Slow Evolving

To determine whether differential destinations of particular proteins would result in characteristic evolutionary rates, we considered different UniProt subcellular localizations ascribed to the *E. coli* and *S. cerevisiae* gene products under study. These represent a curated subset of both RefSeq CDS collections, as only trustable, single-copy orthologous genes with subcellular information were included (n=980 and n=4,104 for *E. coli* and *S. cerevisiae*, respectively; see Materials and Methods). Only genes with annotated subcellular locations were further analyzed as they correlated differently with protein abundance compared to those lacking such annotations (supplementary fig. S1, Supplementary Material online).

**Fig. 1.**
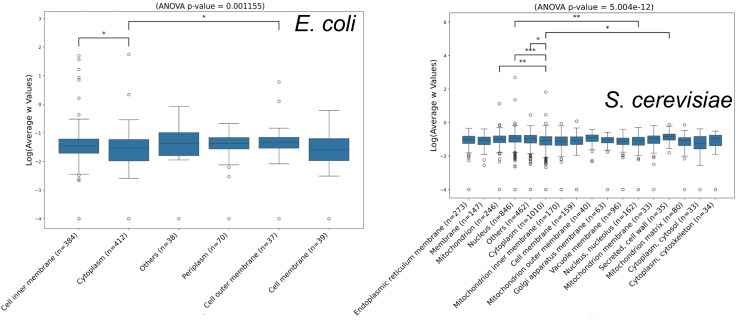
*ω* heterogeneity in different protein subcellular locations. Protein rates of evolution are characteristic of some subcellular compartment in *E. coli* and in *S. cerevisiae*.

Significant general and pairwise differences in means across locations are observed (Fig. 1). Among bacterial compartments, the cytoplasm represented a significantly slow-evolving location, and proteins bounded to inner/outer membranes were fast-evolving (Tukey HSD P<0.05). In yeast, despite a greater diversity of locations available for a protein, we also find cytoplasmic proteins to be much slower-evolving than nuclear (Tukey HSD P<0.001), mitochondrial, nucleolar (P<0.01), and still significantly slower than secreted/cell wall proteins (P<0.05). Our results tentatively agree with the claim that rates are fastest in secreted proteins (Julenius and Pedersen 2006), although our filtering criteria limited the number of secreted orthologous proteins available for comparison. In sum, the above data support a common trend in which a gene is more conserved when the destination of its gene product is the cytoplasm. These results broadly agree with the predictions of the complexity hypothesis (Aris-Brosou 2005), namely that the more peripheral the protein, the faster evolving it is, and we here extend that result to prokaryotes.

### *E. coli* and *S. cerevisiae* Cytoplasmic Proteins have High Abundance

**Fig. 2.**
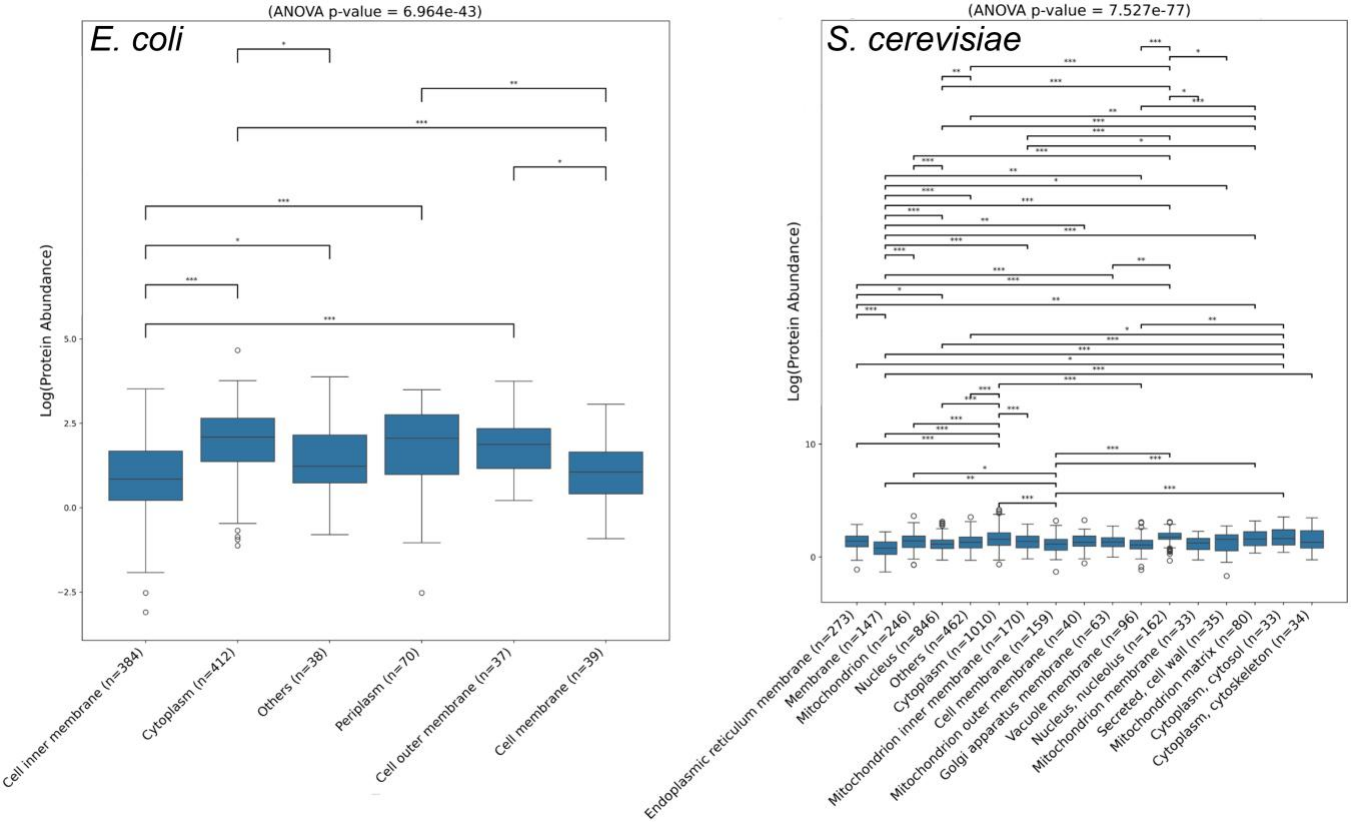
Protein level heterogeneity in different subcellular locations. Almost each compartment has a characteristic protein abundance (regardless the organism), potentially accounting for the observed between-compartment differences in omega.

It could be that this trend in evolutionary rates is predicted from protein abundance differences particular to each compartment. Therefore, we checked heterogeneity in protein abundances across subcellular locations. Our results indicate that differences in protein abundance among compartments are stronger than differences in *ω* in both organisms (Fig. 2). Cytoplasmic proteins tend to be more common than membrane proteins (Fig. 2), as expected given the dimensionality of these compartments (i.e. 3D versus 2D). We conclude that protein abundance must be corrected for.

### Cytoplasmic Proteins are Fast-Evolving when Controlling for Abundance

Having identified the between-compartment differences in both evolutionary rates and protein levels, we questioned the extent to which the abundance of proteins within a compartment explains the evolutionary constraint of the proteins. For the sake of clarity, we collapsed most locations into two parental classes: *Membrane* and *Cytoplasm*. Locations without “membrane” or “cytoplasm” in their name, were collapsed into *Others*. We observe that some classes differ in abundance but not in *ω*. For example, cell inner membrane proteins are on average much less abundant than periplasmic proteins in *E. coli* (Fig. 2a), whereas their rates showed no significant differences—as shown in Fig. 1a. This suggests that there are cell compartment effects that are independent of abundance effects and motivates further scrutiny.

**Fig. 3.**
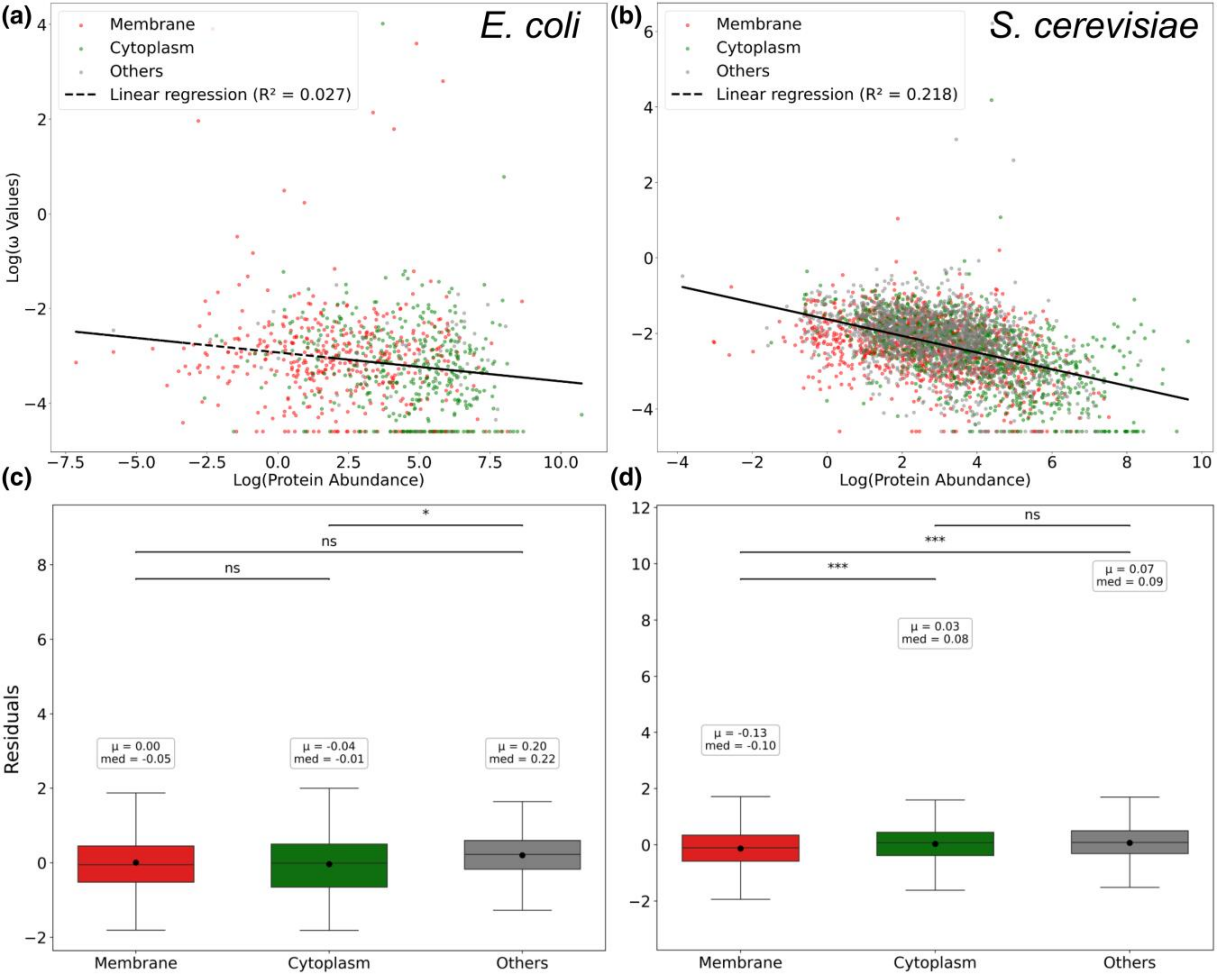
a) Abundance–omega log–log regression in *E. coli*. b) Abundance–omega log–log regression in *S. cerevisiae*. c) Analysis of Variance (ANOVA) analyses suggest that residuals are different between *E. coli* compartments (ANOVA P-value=0.047). d) Residuals are most different among *S. cerevisiae* compartments (ANOVA *P*-value $1.02\times 10^{-12}$). Consequently, membrane and cytosolic proteins have universally different ratios not explained by protein abundance. When controlling for abundance, cytoplasmic proteins evolve faster than membrane ones.

We first sought to recover the classical abundance—*ω* negative correlation. For both species, this was found, but the effect was much stronger in yeast than in bacteria (Fig. 3a and b) for reasons unknown. Given the abundance–rate correlation, we consider the linear regression of log rate of evolution predicted by log protein abundance and determine the residuals for each gene, employing these as our abundance-controlled measures of rates of evolution. Significant differences in residuals were found between cellular location classes (Fig. 3c and d), indicating that protein abundance cannot fully explain protein rates. Importantly, while we observed absolutely higher rates of evolution for cytoplasmic proteins, the regression residuals for membrane proteins were consistently lower, indicating that, surprisingly, when controlling for abundance, cytoplasmic proteins are faster-evolving compared to membrane proteins.

Given the unusual nature of this result, we further tested it with alternative statistical methods. First, we performed binomial tests on *ω* values of cytoplasmic and membrane proteins of the “same” abundance. That is to say, we considered each membrane-associated protein and identified cytoplasmic-associated proteins reported to have similar abundance, defined in terms of a window size. We then consider the nature of the distribution of differences in rate of evolution between the abundance-matched genes. The null expectation is no difference. We find that, regardless the permitted abundance window size, cytoplasmic proteins were faster evolving than membrane proteins (supplementary fig. S2, Supplementary Material online). The size of the effect was greater in yeast. This test, however, excludes much data (e.g. low abundance membrane proteins or highly abundant cytoplasmic ones with no match). Second, therefore, and concerned as to whether the bimodality of log(*ω*) distributions (supplementary fig. S3, Supplementary Material online) might impact the linear regressions, we applied Locally Estimated Scatterplot Smoothing (LOESS) regression analysis to determine residuals. From this, we obtained equivalent results to those seen using linear regression residuals with membrane proteins evolving slower given abundance (supplementary fig. S4, Supplementary Material online). In sum, with no or minimal assumptions about the form of the abundance–omega relationship, cytoplasmic proteins evolve faster than membrane proteins when allowing for protein abundance.

### Differences in Rates of Evolution are not Explained by Essentiality

One possibility to explain the above result is that some other robust correlate, apart from between-compartment protein level is explaining the observed differences in *ω* distributions among subcellular compartments. As dispensability was previously identified as a key covariate when considering extra versus intracellular proteins (Julenius and Pedersen 2006; Liao et al. 2010), we consider this as a variable that should be considered.

**Fig. 4.**
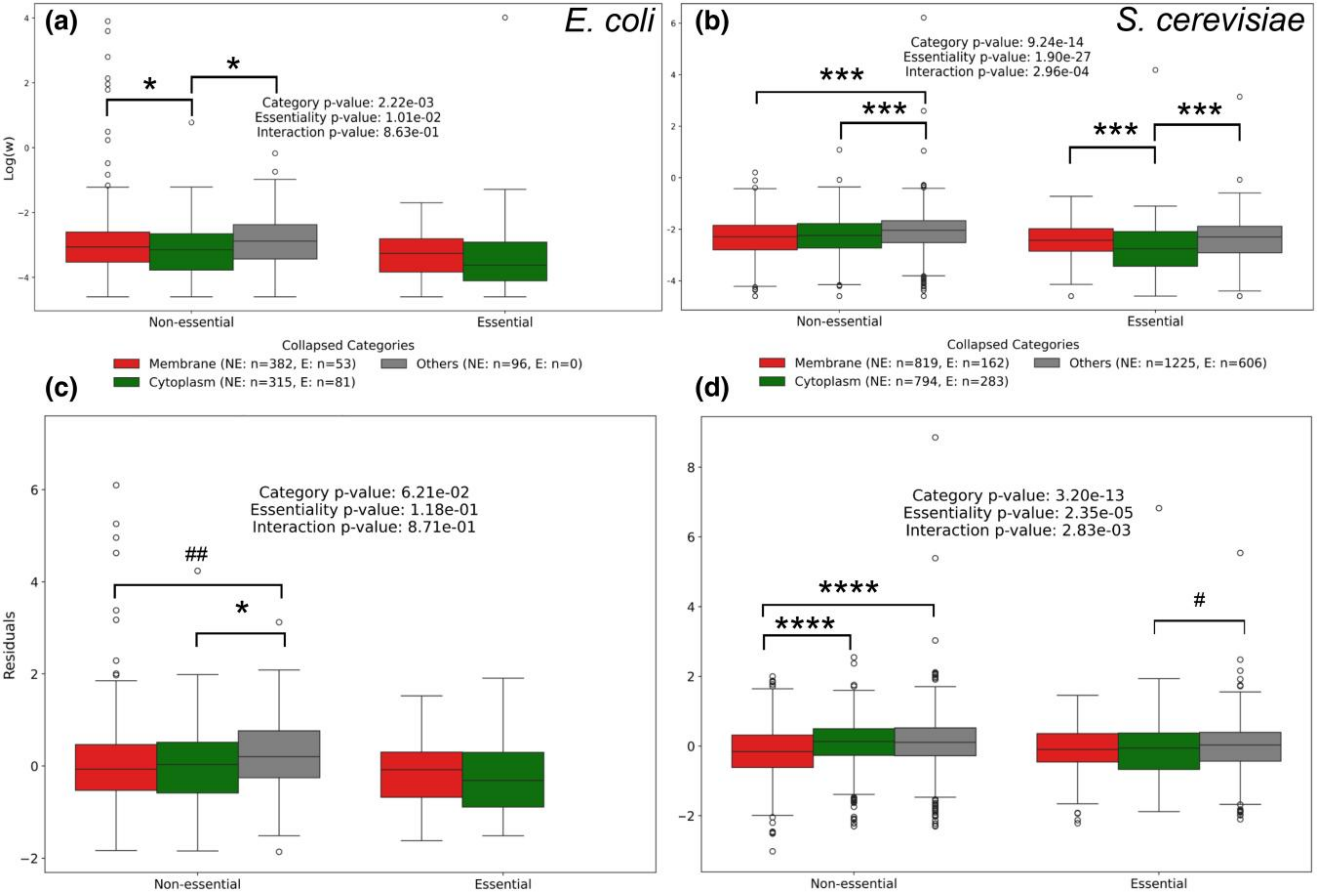
Membrane and cytosolic proteins have different ratios neither explained by essentiality. In a) bacteria and b) yeast, we decouple the previous differences in omega by essentiality. When controlling for abundance (residuals shown in c and d) part of the effect is gone—especially in essential genes—but cytoplasmic proteins then appear to be fast-evolving. This effect appears to be stronger in yeast. The number of data points in panels a) and b) is the same as in panels c) and d). Significance is indicated: ^#^P<0.065; ^##^P<0.058; ^*^P<0.05; ^***^P<0.006; ^****^P<0.0001.

We first observe that *ω* averages between essential and nonessential genes are different (supplementary fig. S5, Supplementary Material online). We then ask whether the rate of evolution within essential and nonessential gene groups showed heterogeneity between cell compartments in *ω*. We find they do (Fig. 4a and b). Any such effect may, however, be owing to abundance differences between the proteins by cell compartment. Thus, we further asked whether the residuals from the overall abundance–omega regression are still different within each essentiality class. We find that there is heterogeneity (Fig. 4c and d) except within the classes essential and nonessential genes from *E. coli* (of which there are relatively few) although, when individual categories are tested, significant differences are observed (supplementary figs. S6 and S7, Supplementary Material online). This result is robust to whether we consider residuals from the parametric or non-parametric regression (supplementary fig. S4, Supplementary Material online).

The above suggests that within both essential and nonessential gene grouping cell location is a predictor of the rate of evolution. However, it does not establish whether the effects are the same within both dispensability classes. To consider this, we determine whether there is any interaction term between essentiality and location. When considering *E. coli* data, we find that location categories consistently explain more *ω* variance than essentiality while allowing for abundance, and that interaction terms are small (Table 1). In *S. cerevisiae*, location accounted for less variation in *ω* compared to essentiality, while the interaction terms contributed minimally. These effects were generally consistent across categorical hierarchies, whether analyzed at the level of specific locations (e.g. “cell outer membrane”) or collapsed parental categories (e.g. “any membrane”). Altogether, these results show the effect of location on rates is mostly distinguished from the effect of a location’s characteristic essentiality.

**Table 1.** (a) Proportion of variance in *ω* explained by each variable based on two-way ANOVAs (sum of squares). Collapsed categories include *Cytoplasm*, *Membrane*, and *Others*; (b) Proportion of variance in residuals explained by the variables (abundance-controlled *ω*)

	(a) *ω*		
Dataset	Essentiality (%)	Location (%)	Interaction (%)
*E. coli* (collapsed loc.)	0.71^*^	1.31^**^	0.03
*E. coli*	0.67^*^	1.72^**^	1.26^*^
*S. cerevisiae* (collapsed loc.)	2.93^***^	1.48^***^	0.4^***^
*S. cerevisiae*	2.90^***^	2.75^***^	0.36

	(b) Residuals		
Organism	Essentiality (%)	Location (%)	Interaction (%)
*E. coli* (collapsed loc.)	0.262	0.597^#^	0.0296
*E. coli*	0.24	1.41^*^	0.14
*S. cerevisiae* (collapsed loc.)	0.452^***^	1.46^***^	0.296^**^
*S. cerevisiae*	0.57^**^	2.83^***^	0.44

Statistical significance is indicated: ^#^P=0.062; ^*^P<0.05; ^**^P<0.01; ^***^P<0.001.

### Integration of Subcellular Location Improves Evolutionary Rate Prediction

Recognizing that subcellular location exerts a genuine and independent effect on protein rates, we investigated the extent to which incorporating location into a predictive model could enhance our ability to explain variability in *ω*. To this end, we performed two factorial ANOVAs mixing the three predictors used—protein abundance, essentiality, and location—for both of our datasets (Table 2). As expected, protein level was the strongest predictor of the variance in *ω* in both cases (1.75% and 18.85% for bacteria and yeast, respectively), although note the absolute weakness of this effect in bacteria. Accounting for location consistently enhanced the prediction of rates, with minimal contributions from the interaction terms—if significant.

**Table 2.** Results of factorial ANOVAs assessing the effects of protein abundance, essentiality, and subcellular location, along with their interactions, on the variability of *ω* in *E. coli* and *S. cerevisiae*

Variable(s)	*P*	Variance explained (%)
*E. coli*		
Essentiality	0.0020	1.00
Subcellular location	0.0082	1.01
Essentiality × subcellular location	0.8944	0.02
Protein abundance	$4.61\times 10^{-5}$	1.75
Protein abundance × essentiality	0.5259	0.04
Protein abundance × subcellular location	0.3763	0.20
Protein abundance × essentiality × subcellular location	0.6348	0.09
Residual	–	95.88
*S. cerevisiae*		
Essentiality	$4.50\times 10^{-29}$	2.47
Subcellular location	$3.26\times 10^{-22}$	1.95
Essentiality × subcellular location	$3.51\times 10^{-5}$	0.40
Protein abundance	$1.62\times 10^{-190}$	18.85
Protein abundance × essentiality	$4.27\times 10^{-6}$	0.41
Protein abundance × subcellular location	$2.11\times 10^{-7}$	0.60
Protein abundance × essentiality × subcellular location	0.1191	0.08
Residual	–	75.24

Interestingly, while in yeast the universal predictor (protein abundance) far exceeded the contributions of essentiality (2.47%) and subcellular location (1.95%), in bacteria this contribution was much closer to the proportion explained by the other factors (≈1%). The latter result suggests that subcellular localization, whilst having a genuine-but-modest impact on yeast compared to protein abundance, may be a relatively stronger (but nonetheless small) determinant of the evolutionary fate for bacterial proteins.

### The Abundance–Rate Correlation is more Pronounced for Cytoplasmic Proteins than Membrane Ones

Above, we have considered variation in the rate of protein evolution as a function of abundance, this being known to be a universal predictor of protein rate of evolution (although the effect is much more modest in our bacterial data than in yeast). Why this correlation is seen is a matter of debate (Drummond et al. 2005; Drummond and Wilke 2008; Cherry 2010; Gout et al. 2010; Yang et al. 2010, 2012; Park et al. 2013). With our result that cell compartment matters, there is a potentially novel explanation related to Simpson’s paradox. To see this, imagine two compartments, let us say membrane and cytosol. If, for example, membrane proteins have low abundance but comparatively high rates of evolution, while cytosolic proteins are the opposite, then the regression of protein abundance predicting protein rate will describe a general negative correlation, even if within both compartment classes there is no abundance–rate correlation. Thus, we ask about the slope of the rate-abundance correlation in each cell compartment class.

**Fig. 5.**
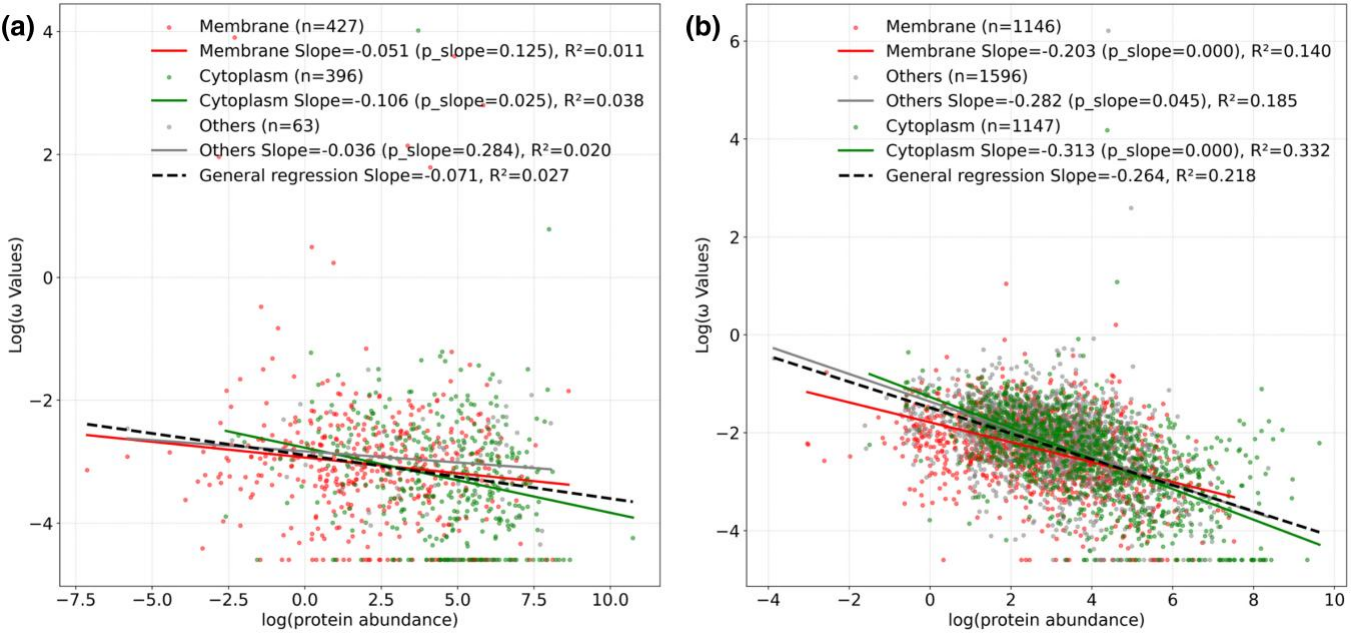
By-compartment regressions in a) *E. coli*; b) *S. cerevisiae*. Membrane and cytosolic proteins have characteristically different slopes, regardless the organism. The explanatory power of protein abundance is universally higher on cytoplasmic proteins.

We find that within each cell compartment, the expected negative correlation is observed (supplementary fig. S8, Supplementary Material online), falsifying the above model. Enigmatically, however, in both *S. cerevisiae* and *E. coli*, membrane proteins have a shallower slope than do cytoplasmic proteins (Fig. 5). Contrary to the above hypothesis, we thus see that to some degree the abundance–rate correlation may be considered to be being masked by the inclusion of membrane proteins.

## Discussion

The hypothesis that different compartments have characteristic rates of protein evolution has withstood all of our falsification attempts. However, unusually, allowing for abundance, the rate of evolution heterogeneity not simply persists but the direction of effects changes: given their abundance, membrane proteins evolve unusually slowly where prior to control they were relatively fast-evolving. This in part reflects the large differences in abundance between proteins that function in different compartments and underscores the importance of covariance control. We are unaware of any case where on control a direction of effect changes rather than simply having an uncorrected effect diminished. The amount of variation in rates of evolution explained by cell compartment appears to be of a similar magnitude to that explained by dispensability. We do not have enough data to arbitrate on the question as to whether secreted proteins have different rates (Julenius and Pedersen 2006; Liao et al. 2010).

The present study also suggests that some results may be phylogenetically commonplace. The slower evolution of cytoplasmic proteins prior to abundance control in both eukaryotes and prokaryotes is to be expected as cytoplasm is 3D while membranes are 2D, which likely impacts protein abundance. As the ratio of the volume of a sphere to its surface area is *r*/3, *r* being the radius, we expect a greater proportional difference in protein abundance between membrane and cytoplasmic compartments for large cells than for small cells. Indeed, in yeast the ratio of the mean abundance of membrane proteins to cytoplasmic ones is 1/15, while in *E. coli* it is 1/5, in line with such a naive model. Assuming all else is equal, a greater proportional difference in the rate of evolution between cytoplasmic and membrane proteins is thus expected when cells are larger according to the simple null that the difference in protein rates of evolution are explained by differences in abundance. In line with this expectation, the cytoplasmic proteins evolve at around half the rate of membrane proteins in *E.coli*, but at one 15th the rate in yeast. The greater expected and observed difference between cytoplasm and membrane in relative abundance, may well also explain why abundance control appears to have a more marked effect in yeast. Here, the corrected rate measures are evidently lower for membrane compared with cytoplasm, while in bacteria the abundance difference is more subtle, the correction in turn being more subtle (Fig. 5). However, the greater proportional difference in protein abundance between the two compartments for large cells than for small cells is only part of the explanation for the difference between yeast and *E.coli*, as the slope of the abundance-evolutionary rate plots is strikingly shallower for *E. coli*, compared with yeast. Nonetheless, such a simple scaling effect model makes for a potentially powerful null model to account for uncorrected rates of evolution as a function of cell compartment. Better models would also factor in complexities such as changes in cell size during a cell’s life, differences in shape (*E. coli* is a rod not a sphere), and the presence of the nucleus, etc.

Assuming the null surface area to volume ratio model, predicting differences in abundance and hence differences in evolutionary rate, is loosely correct, the slow evolution of membrane proteins on control for abundance seen in both species is then the more enigmatic result. This result contradicts the ECH (Aris-Brosou 2005). Slower evolution of intracellular proteins was claimed as evidence (Aris-Brosou 2005) under the supposition that they are more likely to be highly connected with complex systems. However, such a model may also predict slow evolution of nuclear proteins which we do not see. More particularly, this slower evolution now appears to be an artifact of not controlling for abundance. The same model claimed further support from the result that proteins with more interactions are slower evolving. The history of this question is tortuous, not least because experimental method bias can cause a further artifact whereby highly expressed proteins are likely to have a higher proportion of their protein–protein interactions identified (Bloom and Adami 2003, 2004). The cleanest data sets (literature curated for reliable interactions confirmed by independent methods) do not report a correlation between the number of interactions and the rate of protein evolution (Batada et al. 2006, 2007). Similarly, proteins of core metabolism are no more constrained than those of peripheral metabolism (Hahn et al. 2004). We suggest that the ECH is no longer a compelling synthetic framework.

It was previously suggested that differential involvement with immune systems may explain between-compartment heterogeneity (Julenius and Pedersen 2006) with immune-related genes faster evolving, or more broadly, adaptations to ever-changing environments (Sojo et al. 2016). Our data suggest that these are unlikely to be complete explanations. Membrane proteins are those most likely to be accessible to detection by third parties (e.g. host antibody interactions) but are slow evolving when allowing for abundance. Rather, our data support more general models supposing there to be something particular to different cell compartments. We suggest that it is worthwhile to simply consider the different chemical contexts provided by each compartment. One need then merely suppose that there are differences in the viable explorable sequence properties of proteins in different chemical environments. Constraints on folding or interaction with the surroundings are two such possibilities. Indeed, that the membrane-spanning and nonmembrane-spanning sections of the same membrane protein have different rates of evolution, the membrane-spanning sections evolving slower, is in line with the suggestion that the membrane environment is constraining (Jones et al. 1994; Tourasse and Li 2000). One reason that this might be an attractive model is that cellular environments are likely to be conserved and thus each potentially stably associated with characteristic rates. Indeed, while gene essentiality (Rancati et al. 2018) or protein abundance change, the underlying chemical environments of specific compartments are likely to persist. Despite their variable lipid composition across the global phylogeny (Vega-Cabrera and Pardo-López 2017; Hoshino 2024), biological membranes maintain core chemical properties since their inception (Martin and Russell 2003). Likewise, besides the convergent evolution of periplasmic spaces in gram-negative bacteria and yeast, they share key biochemical features. Both are oxidative environments confined between the cytoplasm and the extracellular space, while retaining functions related to cell detoxification and environmental interaction, such as signaling, ion/protein transport, folding by disulfide bond formation, or oxidative catalysis (Stewart 2017; Miller and Salama 2018). What is less clear is whether such a model would be compatible with the inversion of relative rates associated with control for abundance. If membrane proteins are intrinsically more limited in the sequence space that can be easily (by one mutation) explored, how come they are also faster evolving prior to control for abundance?

### Why is Rate-Abundance Correlation different for Cytoplasmic and Membrane Proteins?

Perhaps our most intriguing results concern the nature of the enigmatic abundance–rate correlation. We observe that the slope of the log(protein rate)–log(abundance) correlation is shallower for membrane proteins than for cytoplasmic ones, and shallower for the bacterial trio than for the yeast trio. We are unaware of any systematic analysis of between-taxa variation in the overall slope and regard our result as both enigmatic and worthy of further scrutiny. It may be an artifact of absolute distances, just as *omega* is higher in absolute terms when the species concerned are more closely related (Rocha et al. 2006). Between-taxa comparisons controlling for the synonymous rate would be valuable.

The different slopes for membrane proteins and cytoplasmic ones, suggests that the exclusion of membrane proteins from such considerations may enable better dissection of the causes of this enigmatic correlation, the membrane proteins in effect masking the effect. However, there may be a subtle statistical rationale. Imagine that the relationship is not linear but is instead slightly concave. That is to say, if we take two points at abundances *x* and 2x we can derive the line that connects the means on the *y* axis. If the function is concave, the mean of the *y* axis data at position 3x sits below that expected from projection of this line. If this is the case, then the regression for genes with low abundance (e.g. membrane) will be shallower than that for genes with high abundance (e.g. cytoplasm).

Such an effect may in part explain what is seen in yeast, but does not explain effects in bacteria. We can consider this by analysis of the residuals of the linear model fitted to all data. A concave function is expected to generate a *n* shaped distribution of residuals. We find, however, that in bacteria the quadratic fit is no better than the linear fit of abundance versus residuals (P=0.09), although the quadratic is weakly better in yeast (P=0.007) (supplementary fig. S4, Supplementary Material online). Likewise, if we divide the data into the top half (by median abundance) and consider the regression slopes for the two partitions, in yeast they are different but in bacteria they are not (supplementary fig. S9, Supplementary Material online). In yeast, the effect is in large part because highly expressed membrane proteins are especially slow evolving (supplementary fig. S2, Supplementary Material online). Part of the problem is in the handling of cases where PAML estimates low rates, these all being granted the same non-zero number and appearing as a line of data points in Fig. 3. Given the sensitivity of results to the species concerned (and likely the chosen comparator species), we prefer not to make any strong conclusions. Whether the relationship is not quite linear does not disturb our conclusion that membrane proteins evolve slower when controlling for abundance as our two alternative methods should be unaffected by such issues. Similarly, covariate-controlled results are robust to use of residuals from non-parametric regression (supplementary fig. S4, Supplementary Material online).

### Rejection of ECH is Robust to Alternative Metrics of Protein Abundance

We have presumed that using the accumulated protein abundance data from PaxDB is the best control for gene expression. While we can be confident that it is a better measure than mRNA levels (assuming protein function is the target of selection), it would be foolish to suppose it is perfect. Mass spectroscopy methods of quantification, for example, are known to be affected by sample preparation methodology (Choksawangkarn et al. 2012) with no universally optimal method suitable for both membrane and cytosolic proteins (Choksawangkarn et al. 2012). Currently, however, we see no better measure. Even if PaxDB relies heavily on MS data, our dataset draws from its highest-quality, integrated entries for *E. coli* and *S. cerevisiae*. These include techniques beyond MS—such as immunodetection and single-molecule fluorescence—thus mitigating shared quantification biases.

Nevertheless, recognizing this limitation, we checked if our results can be reproduced with some other estimate of protein abundance that does not rely on direct quantification. Historically, the degree of codon adaptation of a gene has been employed as a proxy of its expression level, under the premise that highly expressed (and highly translated) genes are selected more to employ those codons that match the more abundant tRNA (Sharp and Li 1987b). Since the original Codon Adaptation Index (CAI) (Sharp and Li 1987a), there have been several modified metrics of codon adaptation all of which correlate with expression level. We consider several metrics of codon adaptation for each species and employ the one that best correlates with *omega*, rather than with abundance, this reducing possible circularity as all metrics are derived from abundance measures (or assumptions about which genes are highly expressed). The abundance–omega correlation was found to be strongest using a log odds ratio metric, which may be considered an updated version of CAI (Materials and Methods). Thus, we replaced abundance with this metric in the overall regressions.

In bacteria, we again observe the residuals to be higher in cytoplasm compared to membrane categories, supporting the previously observed inversion in the directionality of the effects (supplementary fig. S10, Supplementary Material online). In yeast, on codon usage mediated control slower evolution of membrane proteins no longer is found, but neither is the faster evolution seen in the uncontrolled analysis (there are no significant differences between classes), i.e. the inversion in rates is not supported (supplementary fig. S10, Supplementary Material online). Nonetheless, we can still reject the ECH given this lack of significant differences between the groups. More generally, the rejection of the prediction of ECH that membrane proteins evolve faster thus appears to be robust to metric to define abundance, although it is worth noting that such results depend on regressions that assume error in the predictor variable (this is optimal given the uncertainty inherent in the utilized metrics of protein abundance).

### Further Caveats

There is most likely also an issue with definitive statements about the role of essentiality as, while treated as a simple binary classifier, in practice, it is more subtle than this. Genes can, for example, be essential in one environment/context and not another. Thus, while we observe that dispensability explains about as much variation in rate of evolution as cell compartment, this must come with a necessary caveat. That both metrics are categorical, however, means that problems with reduced influence associated with categorical variables, as opposed to continuous, are avoided.

A further caveat is that the genes that we could not classify by cell location do not necessarily evolve by the same rules. However, in both species, the cytoplasmic and membrance proteins constitute a good proportion of all genes. In *E. coli*, out of 980 single-copy orthologous proteins, 46.94% were membrane-bound and 42.04% cytoplasmic. Similarly, in *S. cerevisiae* (4,104 genes), the proportions were 26.34% and 27.95%, respectively. We can thus conclude that a significant portion of the genome evolves differently depending on the subcellular destination of its products. We have also presumed that the UniProt classifications of cell location are both correct and unambiguous. Proteins that shuttle between cell locations but are classified as having one would be logically problematic, but in practice should just add noise to the analysis rendering it conservative.

## Materials and Methods

### Orthologous Sequence and Metadata Retrieval

RefSeq genomic coding and protein sequences were downloaded from NCBI (2024 October 18) for the following species: *E. coli* K-12 (GCF_000005845.2), *Escherichia fergusonii* (GCF_020097475.1), *Salmonella enterica* (GCF_000006945.2), *S. cerevisiae* S288C (GCF_000146045.2), *Saccharomyces paradoxus* CBS432 (GCF_947241705.1), and *Saccharomyces mikatae* IFO 1815 (GCF_002079055.1). Bacterial and yeast proteomes were independently processed with OrthoFinder (version 2.5.5) standard configuration (Emms and Kelly 2019). We limited the posterior analysis to single-copy genes in order to be conservative. The species trees utilized for posterior d*N*/d*S* calculations were directly obtained in this step from the OrthoFinder output (Emms and Kelly 2018).

A custom data-mining software was used to retrieve subcellular location and gene information for each identified ortholog from the UniProtKB JSON files. To this end, we matched the RefSeq identifiers with unique gene names. Abundance data for *E. coli* and *S. cerevisiae* proteomes were manually downloaded from PaxDB (https://pax-db.org/) and then merged with the metadata matching the UniProt retrieved gene name for each ortholog. Subsequently, we retrieved the dispensability data for *E. coli* K-12 from the Keio collection (Yamamoto et al. 2009) (https://shigen.nig.ac.jp/ecoli/pec/download.jsp), and for *S. cerevisiae* from the yeast phenome DB (Turco et al. 2023) (https://yeastphenome.org/)

### Alignment of Orthologous Sequences

Putative ortholog protein sequences were matched to their corresponding RefSeq genomic data using their protein IDs. Subsequent three-way alignments were performed for each orthogroup corresponding to single-copy genes using MAFFT (version 7.526) standard configuration for protein sequences (Katoh and Standley 2013). Codon alignments for each orthogroup were obtained from the protein and nucleotide alignments using PAL2NAL (version 14) software (Suyama et al. 2006).

### Evolutionary Rate Estimation

Rather than considering protein evolutionary rates in a temporal sense, we determine for each gene the number of protein changing substitutions (per nonsynonymous site) per synonymous site change (alias $K_{a}/K_{s}$, d*N*/d*S*, omega). Such values are likely to be correlated with protein rate per unit time but additionally allow for variation in background rates of evolution. We calculated protein rates *ω* on each of the alignments using PAML (version 4.10.7) software (Yang 2007). The analysis was performed under the GY94 codon substitution model (Goldman and Yang 1994), with codon frequencies estimated under the F3x4 model, which calculates nucleotide composition at each codon position. The tree topologies were forced to make these calculations, with *E. coli* and *E. fergusonii* the ingroup, *S. enterica* the outgroup in bacteria, and *S. cerevisiae* and *S. paradoxus*, the ingroup with *S. mikatae* the outgroup in yeast. We considered *ω* for the branch from *E. coli* to the ingroup common ancestor, and for the branch from *S. cerevisiae* to the ingroup common ancestor. This was done to ensure that the evolutionary rates accord as closely to the focal species’ parameters (cell location, protein abundance, essentiality, etc.) as possible.

### Database Curation

We started with 2,385 (bacteria) and 4,967 (yeast) putative single-copy orthologs. In order to have a cleaner dataset, we first observed the d*S* distributions (from the focal species to the ingroup common ancestor). As the histograms of d*S* showed some disparate values, we further performed an interquartile test to delimit the upper limit and filter out dubious orthologs in the bacterial dataset (>0.93 d*S E. coli* or >1.35 d*S* in *Escherichia fergusonii* were filtered out). Additionally, as the samples with and without subcellular location information were not random (i.e. they exhibited significantly different slopes in the abundance–omega correlations, supplementary fig. S1, Supplementary Material online), we further limited the study to single-copy orthologs with subcellular and essentiality information. We ended up with 885 and 3,889 single-copy genes for bacteria and yeast, respectively. The input data, workflow, and code utilized in this study are publicly accessible at 10.5281/zenodo.14720111.

### Statistical Analyses

We performed Deming bivariate regressions on abundance and evolutionary rate levels because normal errors can be assumed for both log-transformed variables (Figs. 3 and 5). The distribution of evolutionary rates is bimodal due to—unlikely—null values, thus we take a conservatory approach and included a pseudo-count in the log transformation. Its value was set to 1/min(d*S*) ≈ 0.01 to ensure that it does not alter the overall distribution of data points and to limit the biases in the subsequent regressions. Tables 1 and 2 were obtained from multiple regression analysis including first-order interacting terms. In Fig. 5, the significance of regression parameters for subcellular location data was obtained after comparing the observed value with $10^{4}$ randomizations under the null hypothesis of subcellular location independence in each case, controlling for group size. Binomial tests (supplementary fig. S2, Supplementary Material online) were performed by pairing each membrane protein with the closest-abundance cytoplasmic protein within a fixed window, and testing whether $\omega_{\mathrm{cytoplasm}}>\omega_{\mathrm{membrane}}$ occurred more often than expected by chance.

To quantify codon usage bias to avoid systematic biases of direct protein quantification, we employed three metrics: (i) the CAI (Sharp and Li 1987a), (ii) a tRNA adaptation index incorporating refined tRNA expression estimates (Wei et al. 2019), and (iii) a log odds-based measure of codon bias designed to capture patterns associated with translational efficiency (Lewin et al. 2023). Method (i) employed the original scoring tables of Sharp and Li (1987a) for *S. cerevisiae* and *E. coli*, linking the CAI values to orthologs matched to PaxDB protein abundances. Method (ii) considered deviation from null in the usage of the optimal codon in any given codon block, the optimal codon being defined by the intersection of enrichment patterns in highly expressed genes and tRNA transcriptomic abundance. In any given gene, for each degeneracy class, the mean (O−E)/E was determined where *O* is the count number of optimal codons observed and *E* is the number of synonymous codon blocks divided by their degeneracy. Weighted means across degeneracy classes provided a final score per ortholog. For the log odds approach (iii), we considered all annotated orthologs and extracted the central 10 codons from each gene to focus on elongation-associated biases while avoiding 5′ end confounds. Genes were ranked in quartiles by protein abundance, and codon counts were separately tallied for the top and bottom quartiles of this ranking. For each codon, we computed the log odds ratio as the natural logarithm of its relative frequency in high-abundance versus low-abundance genes (top versus bottom 25%). Individual gene scores were obtained by averaging the log odds ratio values across their breadth of codons. All approaches are calculated by avoiding the 20 first codons as they are enriched by AT and are not obviously under the same translational selection as the rest of the gene.

## Supplementary Material

evaf126_Supplementary_Data

## Data Availability

Further detailed step-by-step explanation of statistical procedures and packages used as commented python code, as well as all bacterial and yeast datasets utilized in this manuscript are available at 10.5281/zenodo.14720111.
