# Supplementary material for: Cell Compartment is a Predictor of Protein Rate of Evolution, but not in the Manner Expected: Evidence Against the Extended Complexity Hypothesis
Source: Genome Biol Evol. 2025 Jun 21;17(7):evaf126. doi: 10.1093/gbe/evaf126 (PMC12271741; doi:10.1093/gbe/evaf126)
Supplement: evaf126_Supplementary_Data [file evaf126_supplementary_data.pdf]

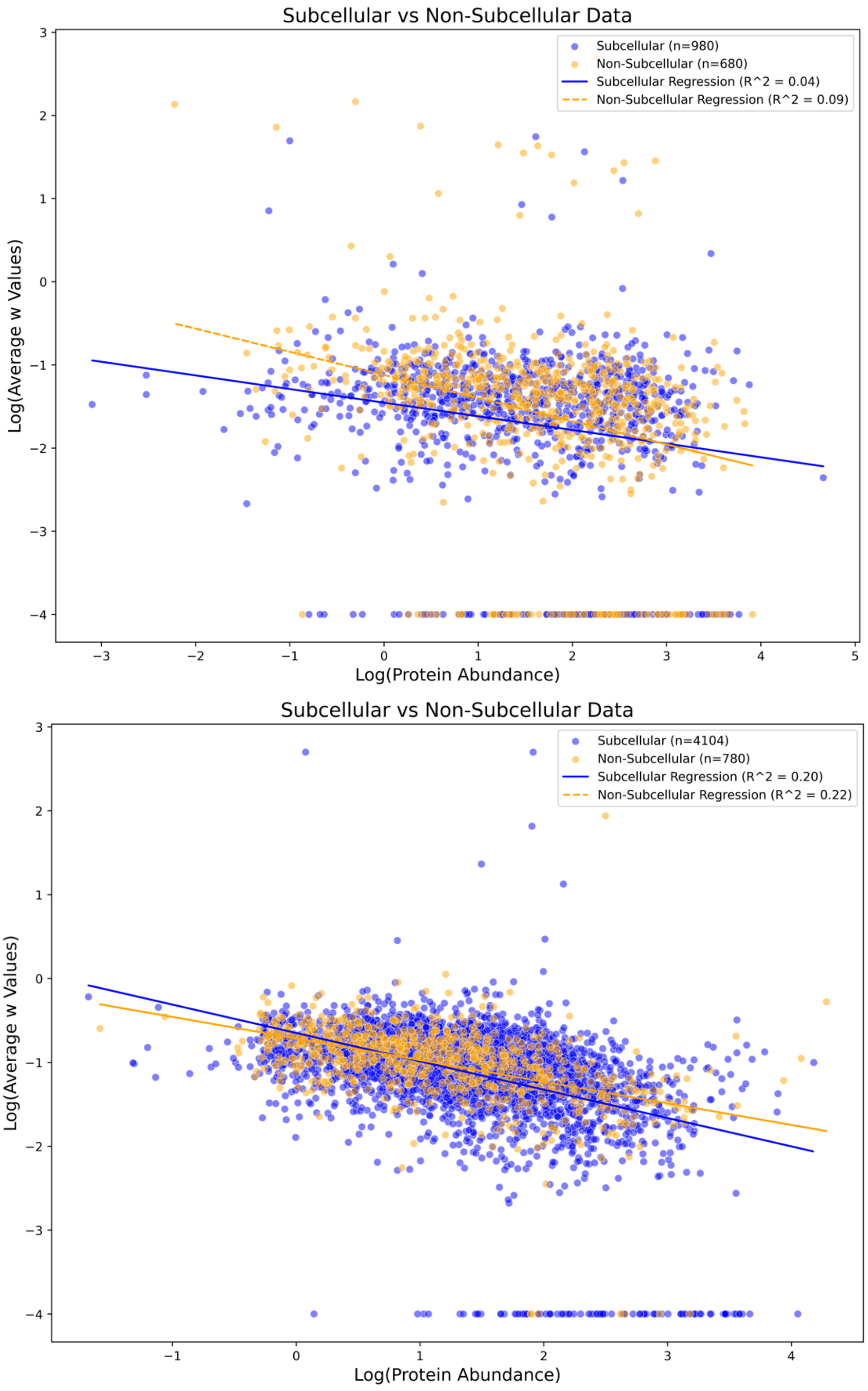

Figure S1. Log-log regressions of E. coli and S. cerevisiae evolutionary rates vs protein abundance on single copy orthologous genes. We do observe that samples with and without subcellular location information in UniProt are not random, and annotated proteins are probably biased by limitations related to the annotation technique employed. To be conservative, we tested our hypothesis only in the sample with subcellular information available.

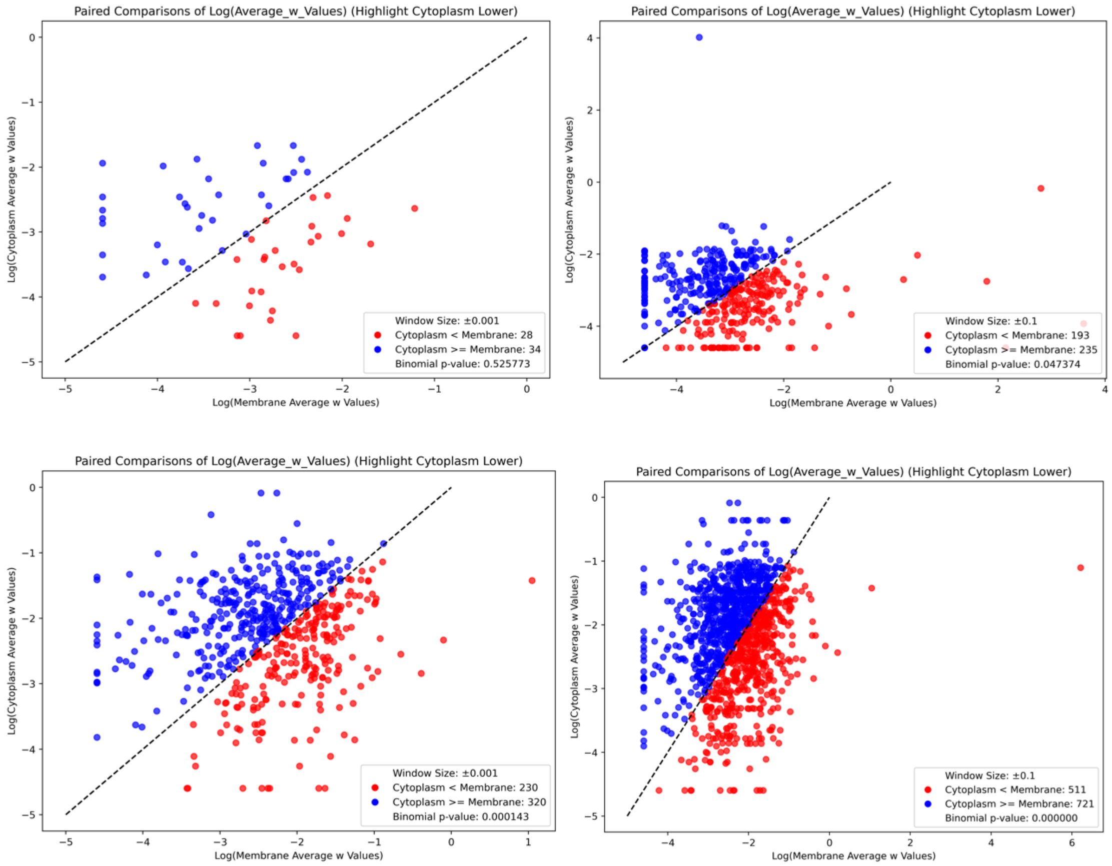

Figure S2. Binomial tests on omega distributions restricted to equivalent omega abundance windows. Left, *E. coli*; right, *S. cerevisiae*. These show significant differences among cytoplasm and membrane. We started making list of dN/dS pairs with each membrane protein, found closest cytoplasmic by abundance, and accepted the pair if falling inside the fixed window. This result is a conservative estimate of the difference of dN/dS means between these two locations controlling for abundance. We observe that cytoplasm is a fast-evolving compartment regardless the abundance window size, and that this effect is stronger in yeast.

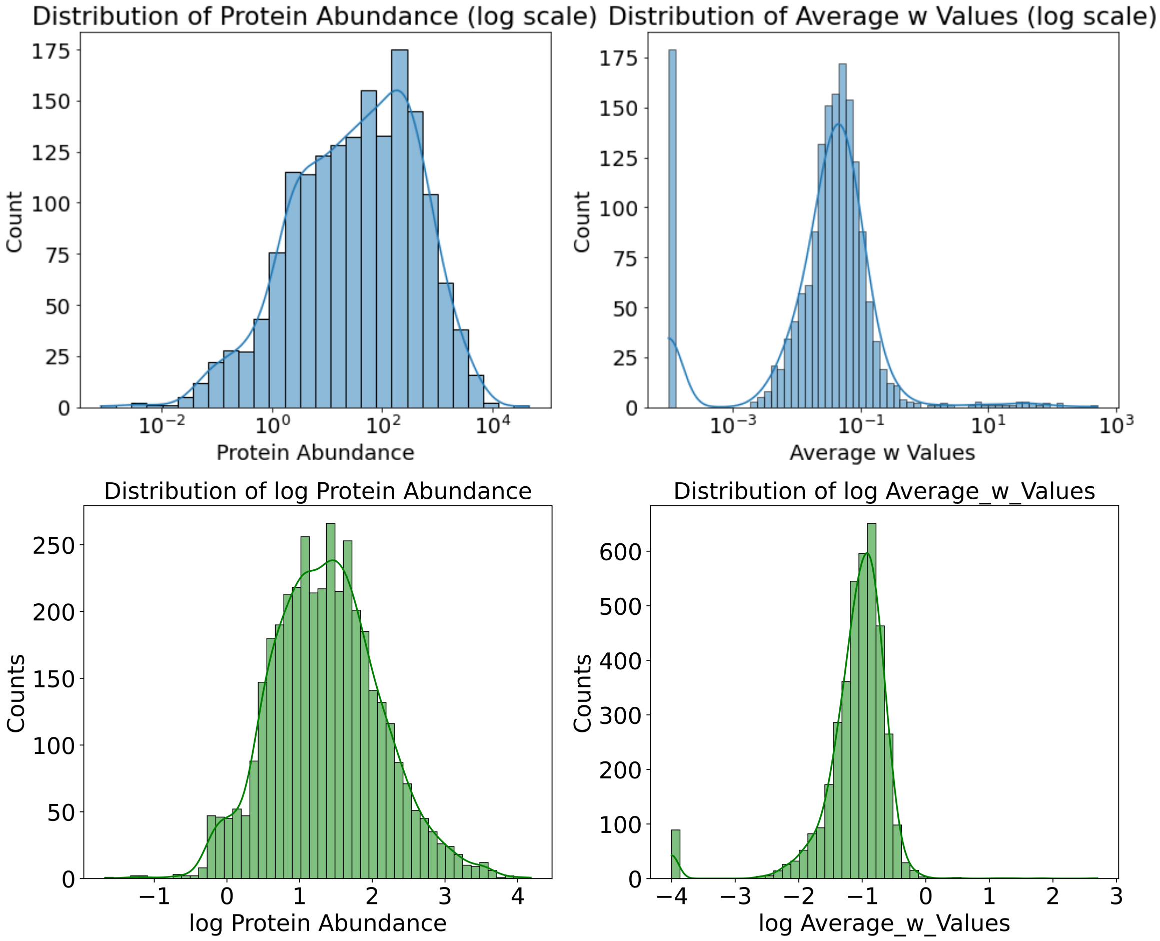

Figure S3. Distributions for log(protein abundance) and log(omega); blue: *E. coli*, green: *S. cerevisiae*. While log(protein abundance) displays normality, log(omega) is a bimodal distribution in both cases.

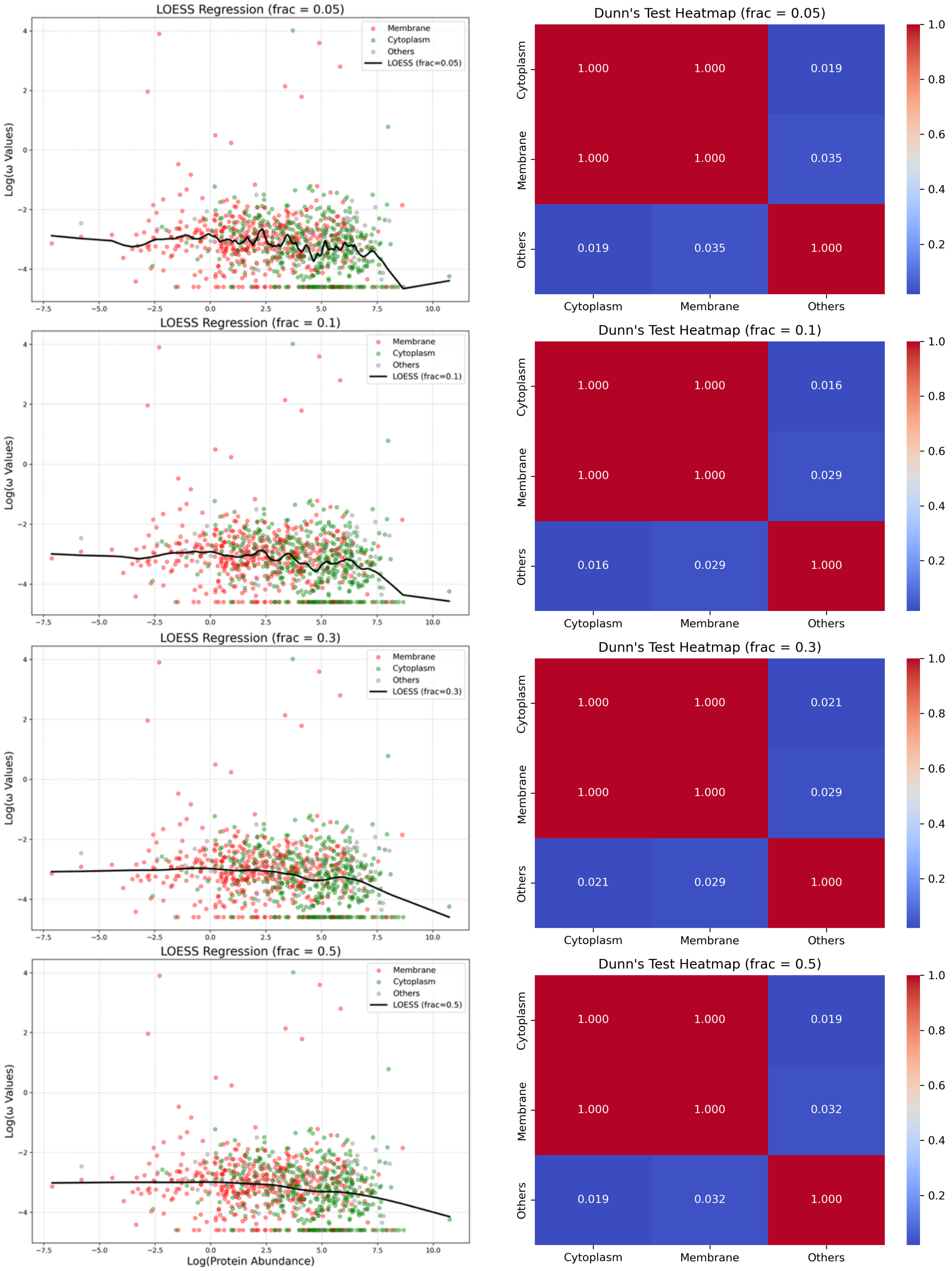

Figure S4.1. LOESS regressions (*E. coli*). We wondered if bivariate regressions were not capturing the nature of our data accurately. However, residuals show the same trend with the bivariate linear regressions, suggesting that our interpretation is not contingent to the kind of analysis employed. We show *p*-values and regressions with different frac (smoothness) parameters, all of them consistent with our interpretation.

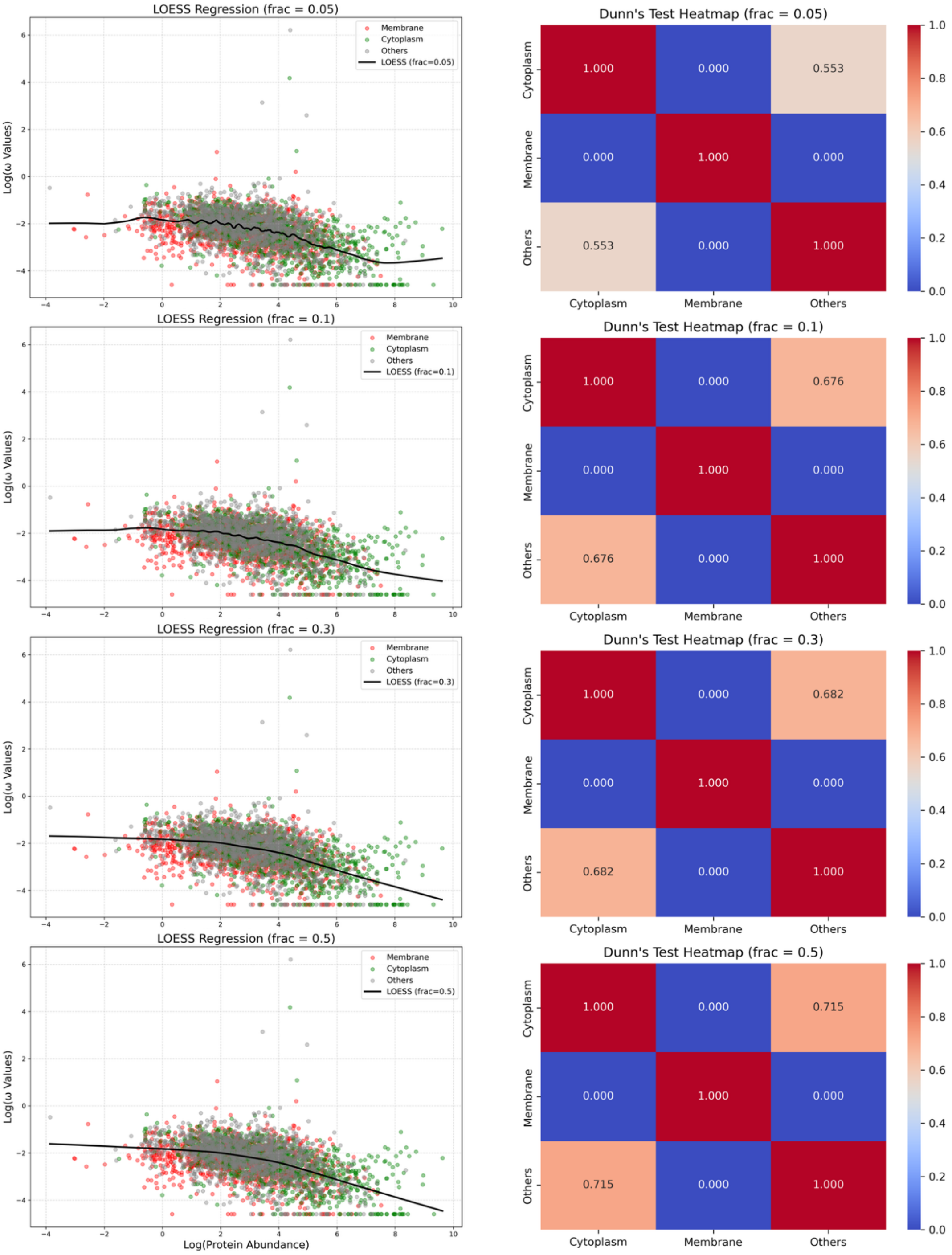

Figure S4.2. LOESS regressions (*S. cerevisiae*). We wondered if bivariate regressions were not capturing the nature of our data accurately. However, residuals show the same trend with the bivariate linear regressions, suggesting that our interpretation is not contingent to the kind of analysis employed. We show *p*-values and regressions with different frac (smoothness) parameters, all of them consistent with our interpretation.

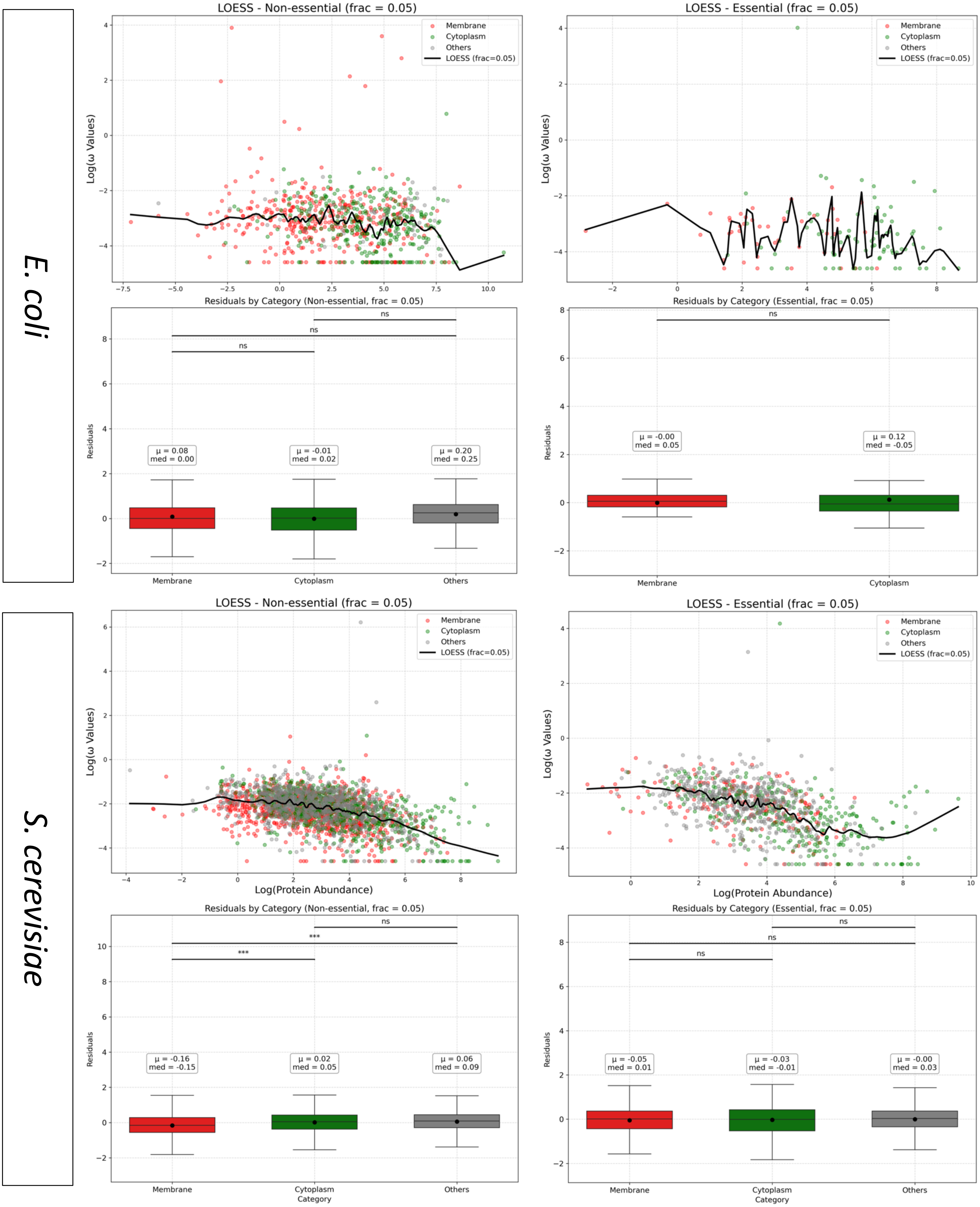

Figure S4.3. LOESS regressions (essentiality tests). We used the least smooth parameter.

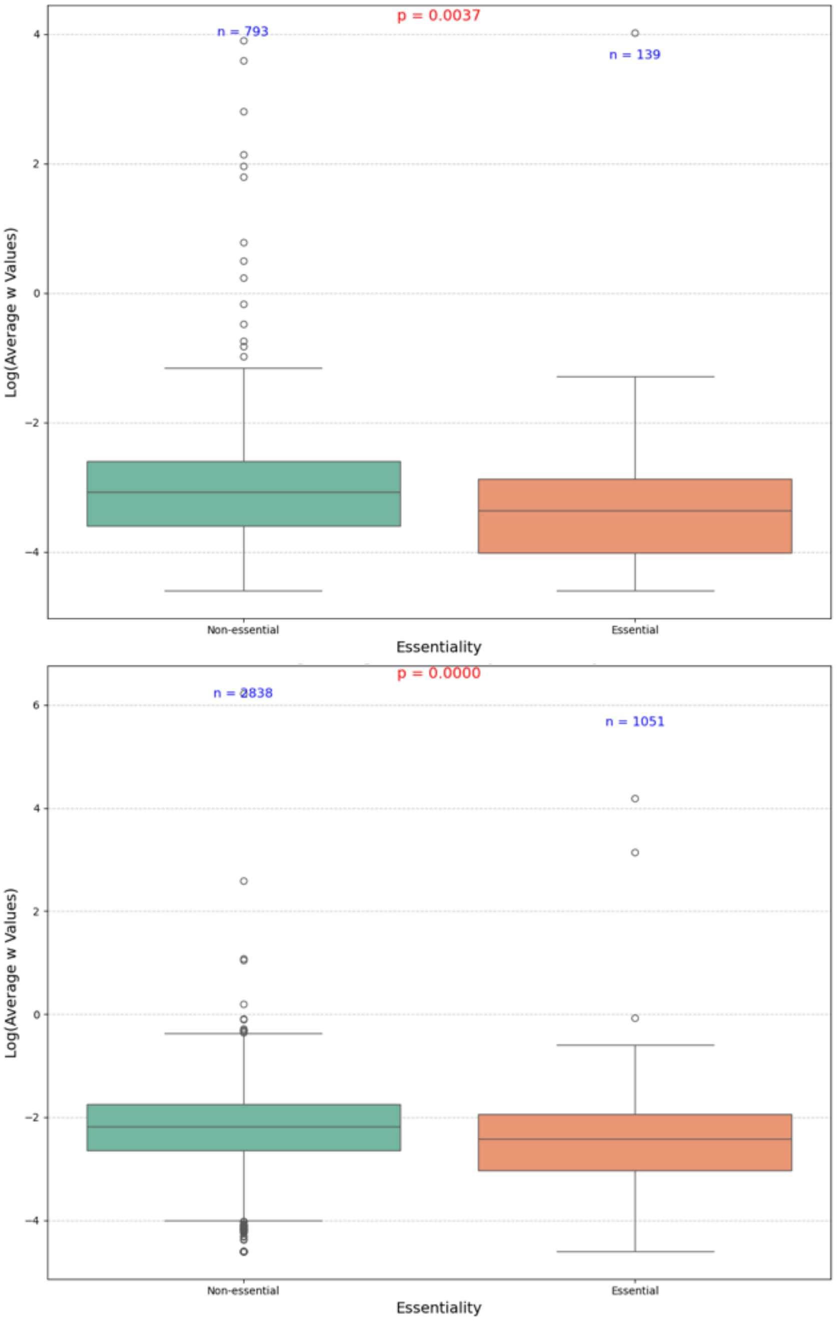

Fig. S5. Distribution of log(omega) values segregated by dispensability classes. Essentiality predicts differences in omega in both *E. coli* (above) and *S. cerevisiae* (below). Essential genes, when uncontrolled by abundance, are significantly more constrained.

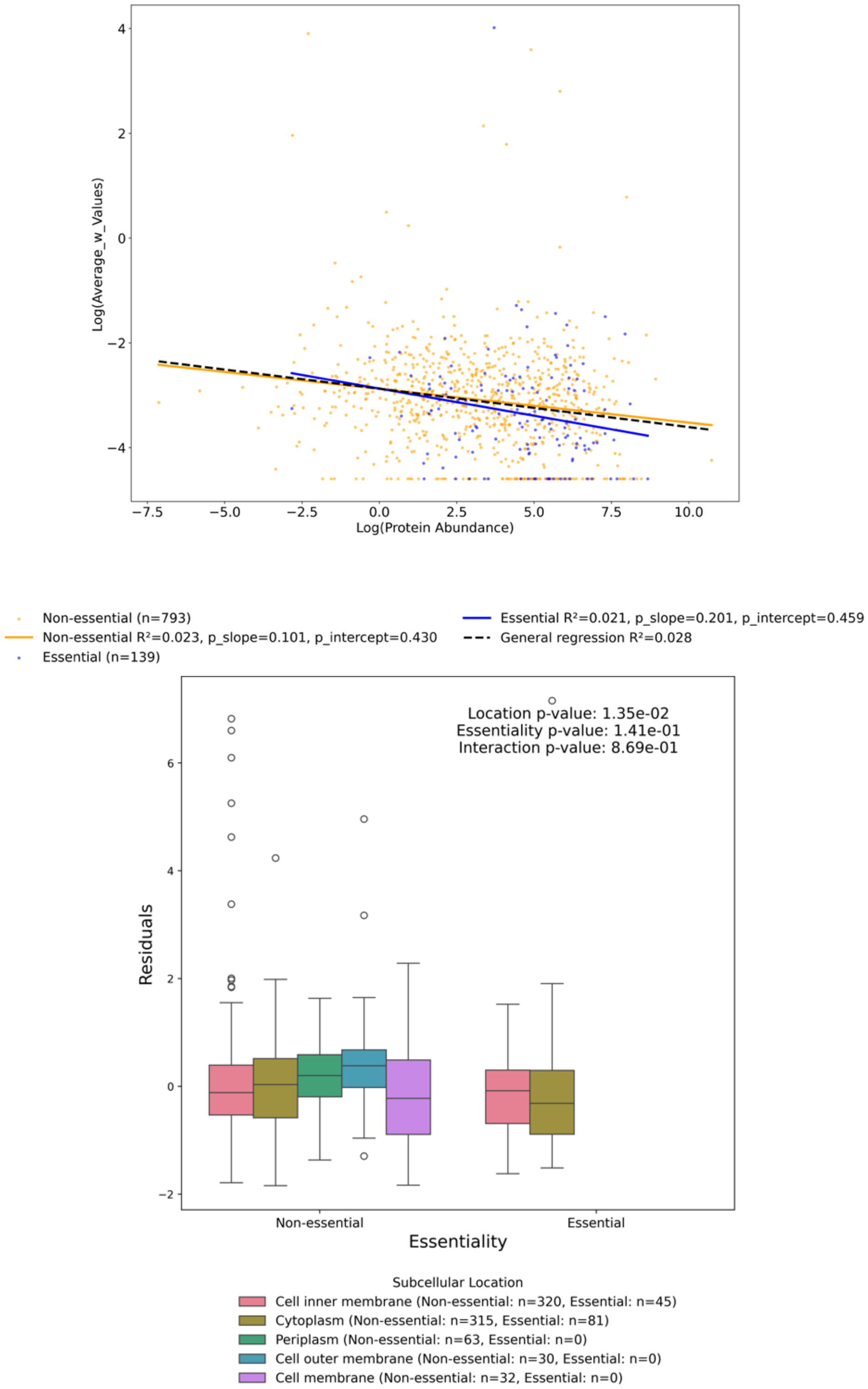

Figure S6. Dispensability-wise regressions and omega distributions by specific uncollapsed locations in *E. coli*. Essential and nonessential classes show different slopes in *E. coli*, although nonsignificant. Uncollapsed location classes (discriminated by essentiality class) show different trends with respect omega when controlled by abundance.

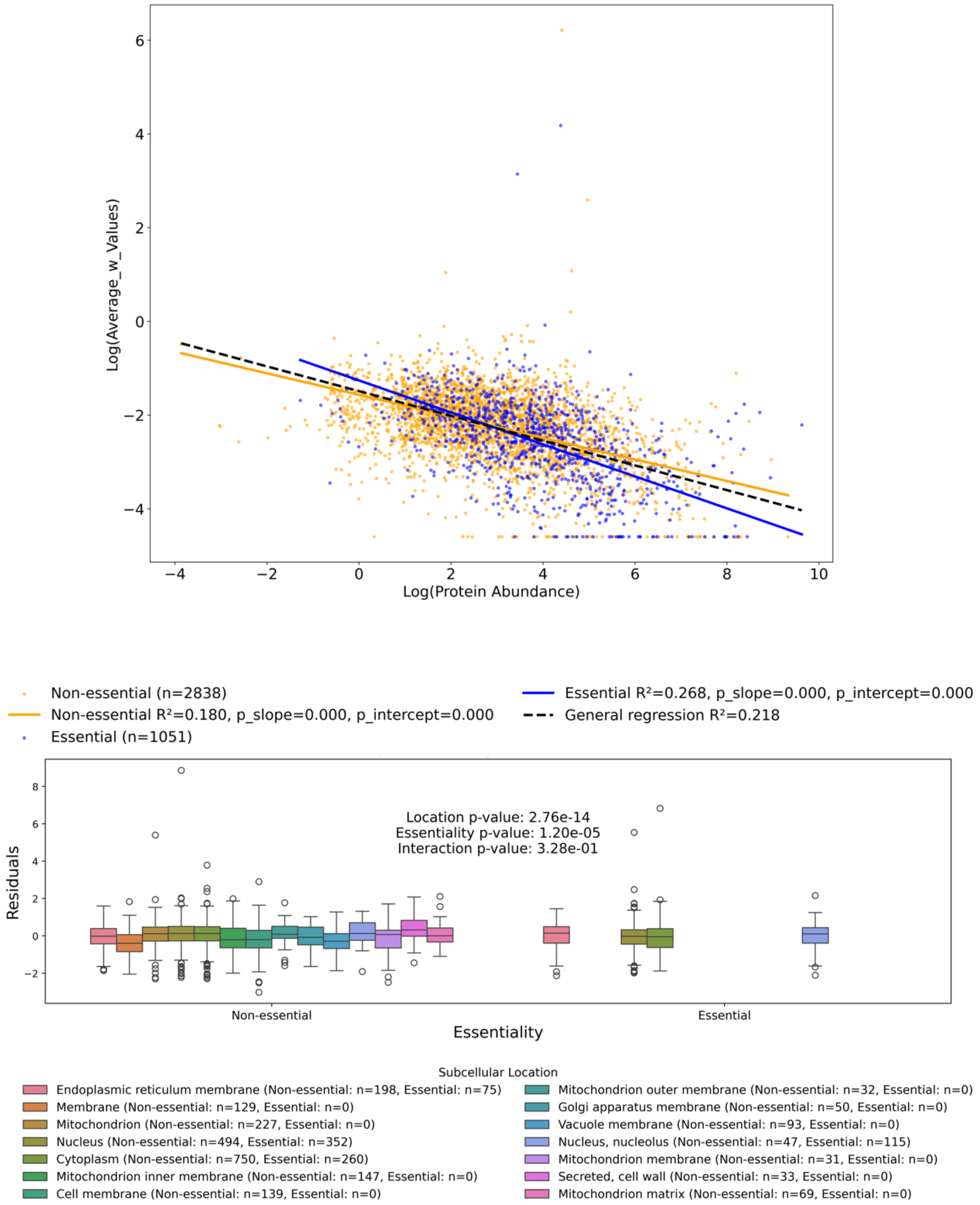

Figure S7. Dispensability-wise regressions and omega distributions by specific uncollapsed locations in *S. cerevisiae*. Unlike in bacterial orthologs, the slopes are significantly different in yeast, and the abundance correlation better within essential genes. The between-compartment omega (discriminated by essentiality class) is also heterogeneous even when controlled by abundance.

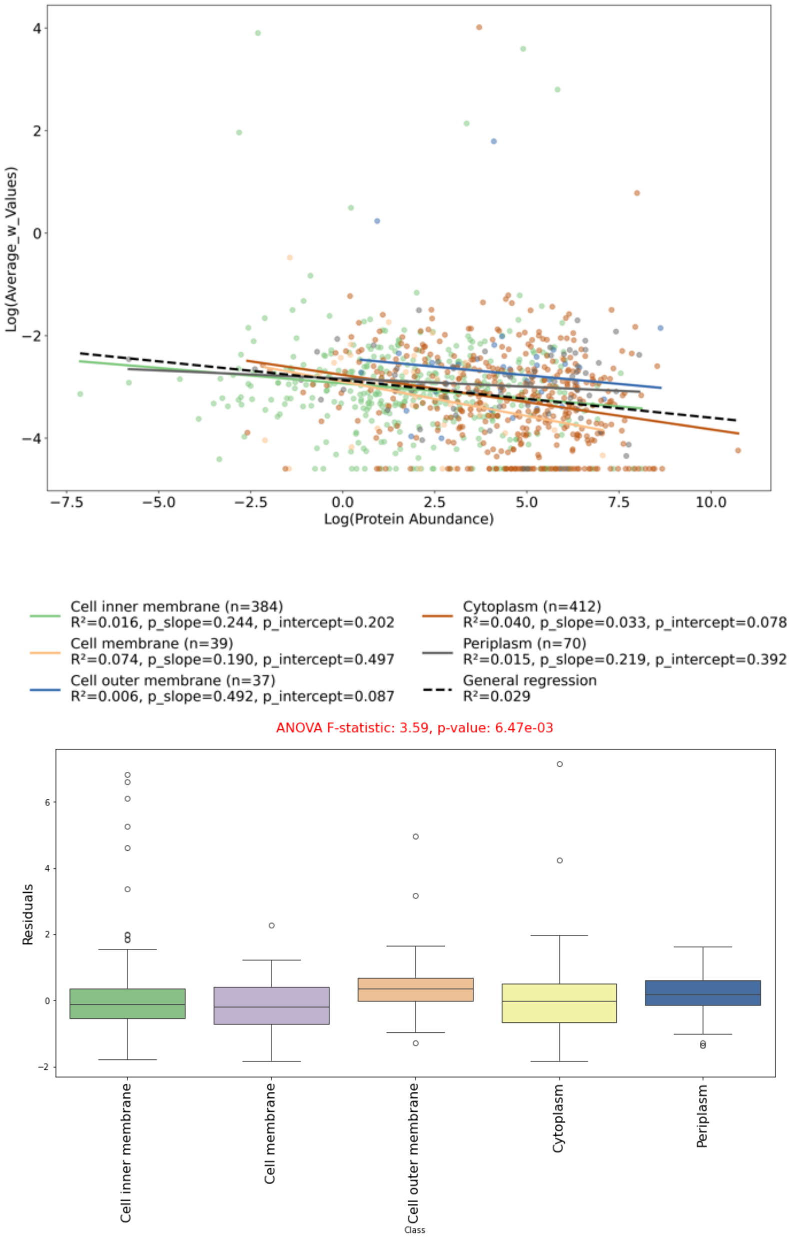

Figure S8.1. (bacterial compartments). Log-log regressions of rates vs abundance across compartments. These are uncollapsed by membrane/cytosol categories – unlike those showed in the main text. Every category with >30 data points was plotted. We do observe different trends that represent departures from the general regression. As in the collapsed categories, distribution of residuals of these lesser-hierarchy compartments remains significantly different when controlling for abundance.

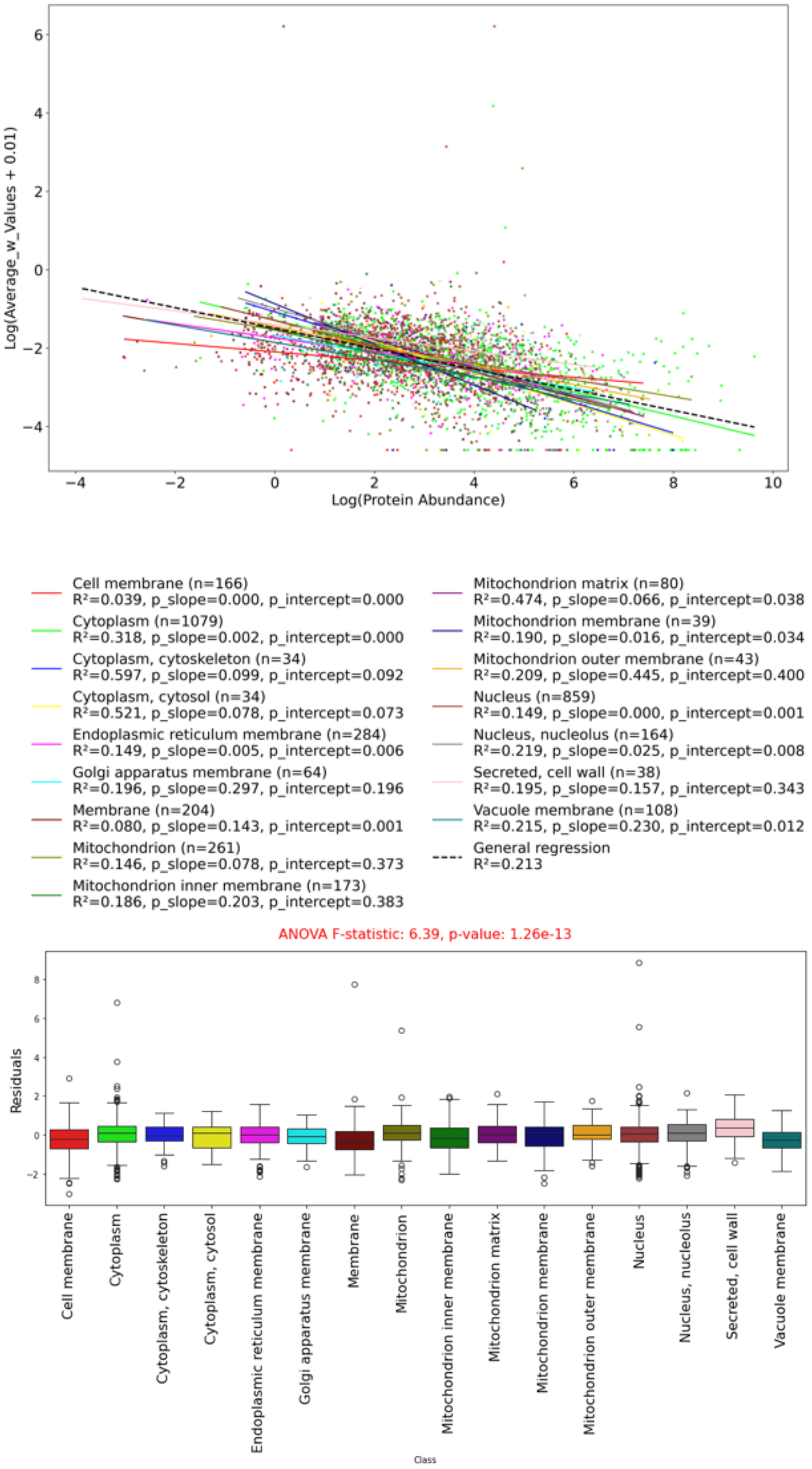

Fig. S8.2. (Yeast compartments)

See S8.1. caption

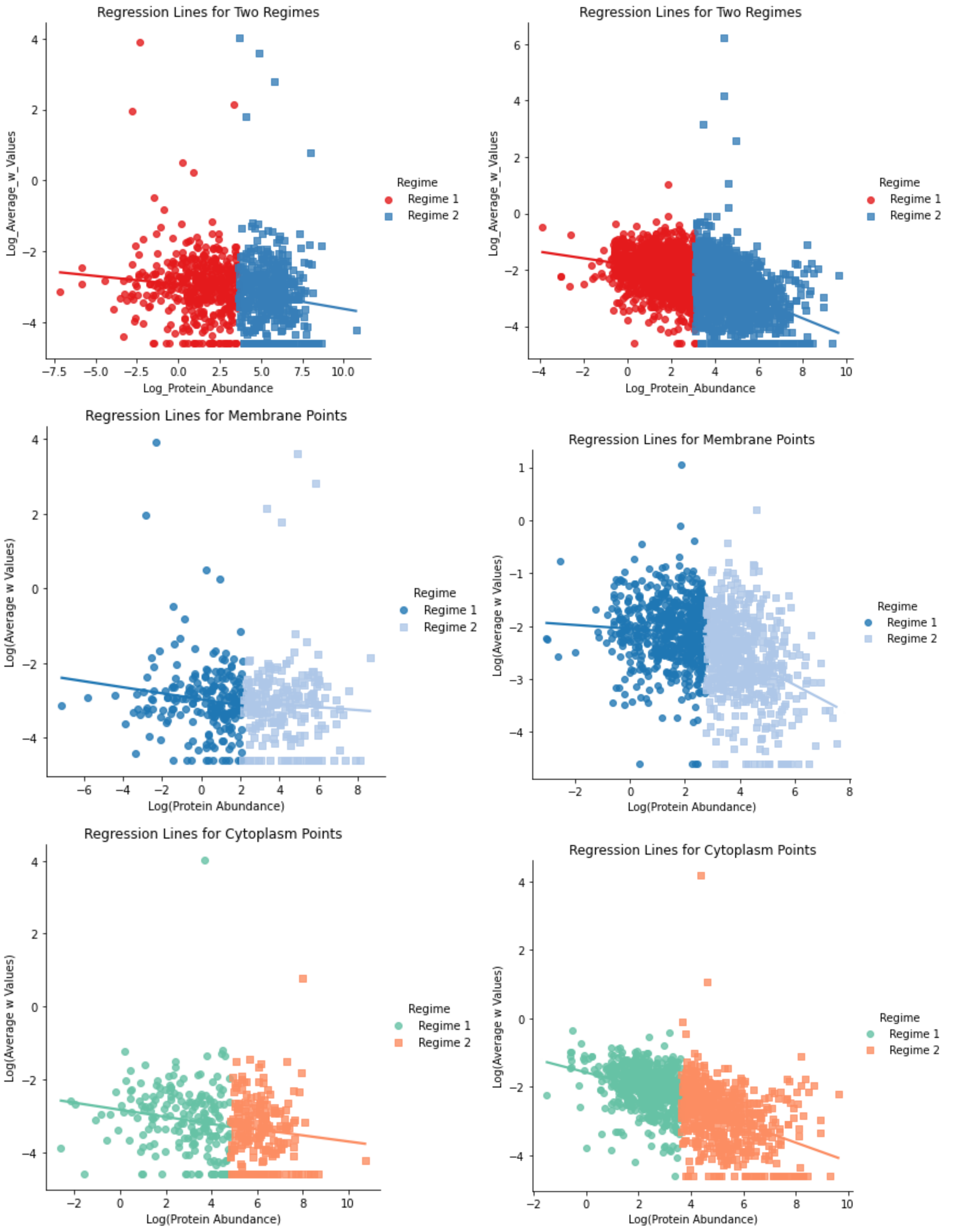

Figure S9. Two-regime bivariate regressions. Left: bacteria; right: yeast data. We further considered if the observed between-compartment differences in slopes could be attributed to characteristic abundance differences. We calculated the median of log(abundance) for (i) all data points, (ii) only membrane, and (iii) only cytoplasm, each for *E. coli* and *S. cerevisiae*. In this manner, we have two regimes to compare (low abundance, or regime 1, and high abundance, or regime 2). Significant differences in slope between regimes were only detected in data of *S. cerevisiae* (right panels), and only in membrane data points (middle). Importantly, correlation is much stronger in regime 2 than in regime 1 of the membrane class (-0.325 vs -0.058 n.s.). When considering the overall regime (1+2) for the membrane class, the correlation is the weakest among classes (-0.374 vs -0.572 of the cytoplasm class, and -0.467 for the whole dataset), and the slope, the flattest (Fig. 5 of main).

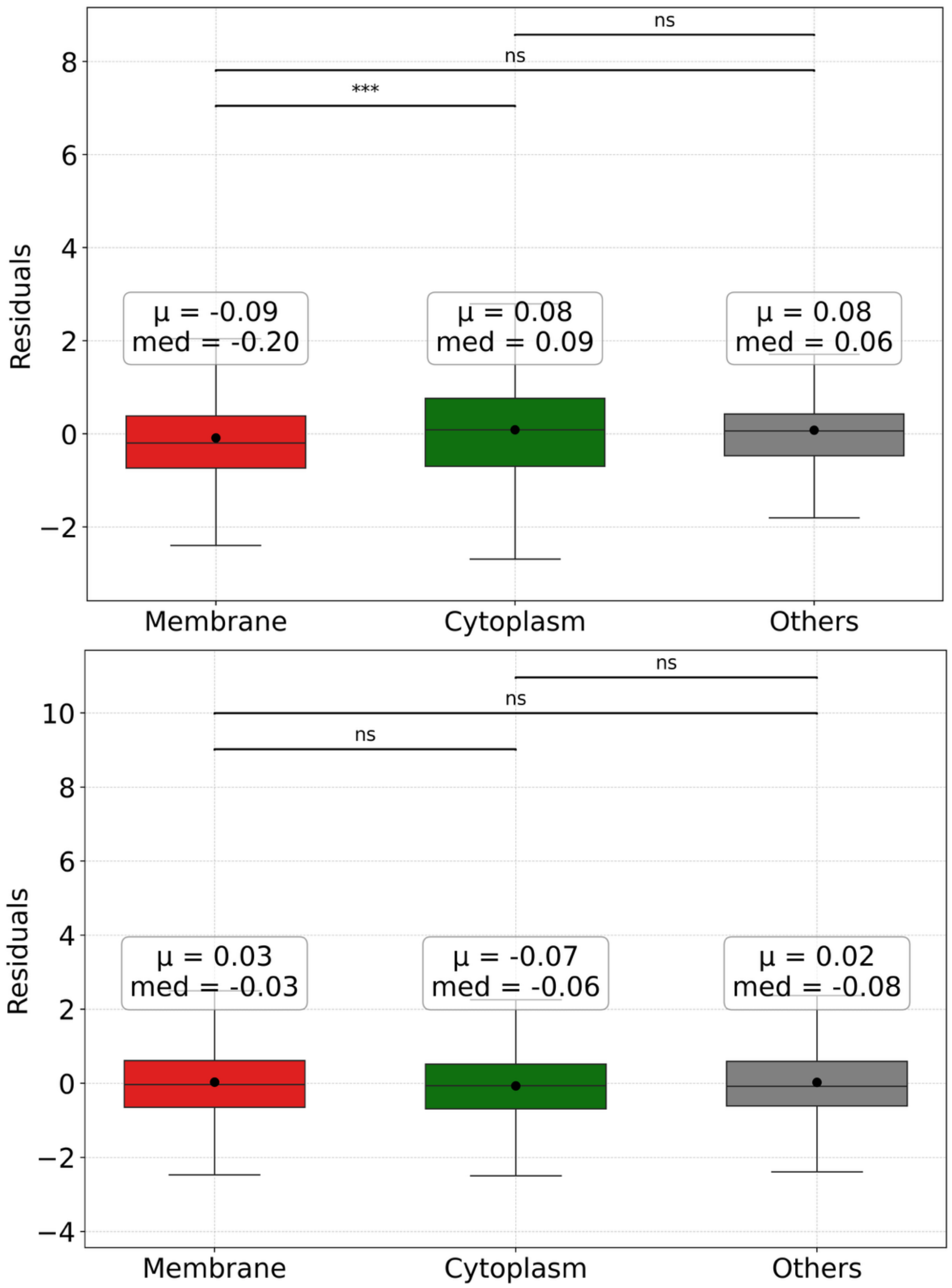

Figure S10. Residuals from codon log-odds ratio-controlled regressions for bacterial and yeast proteins by subcellular location. Above: *E. coli*, below: *S. cerevisiae*. Residuals reflect deviations from the general relationship between ω (dN/dS) and log-odds scores of codon enrichment in high-abundance proteins. Since three tests (Shapiro-Wilk, Anderson-Darling, and Kolmogorov-Smirnov) strongly reject the null hypothesis of normality of the residuals, we used a nonparametric test to capture the difference among these distributions. Kolmogorov-Smirnov and Monte Carlo tests show that cytoplasmic proteins in bacteria (green, above) have significantly lower residuals than membrane proteins (red; D = 0.1575, p = 6.31e-05). This difference is however nonsignificant in yeast (D = 0.0412, p = 0.278). Membrane and "other" proteins do not differ significantly in either organism (bacteria: D = 0.1593, p = 0.110; yeast: D = 0.0295, p = 0.591). Similarly, cytoplasmic and "other" proteins do not differ significantly (bacteria: D = 0.1522, p = 0.144; yeast: D = 0.0431, p = 0.162).
